# Persistent Respiratory Symptoms Post-COVID-19 in a Non-Hodgkin Lymphoma Patient: A Case Report

**DOI:** 10.7759/cureus.91885

**Published:** 2025-09-09

**Authors:** Ilias E Dimeas, Styliani Papra, George Dimeas, Ioannis Pantazopoulos, Zoe Daniil

**Affiliations:** 1 Department of Pulmonology, University Hospital of Larissa, Larissa, GRC; 2 Department of Internal Medicine, AHEPA University General Hospital, Thessaloniki, GRC; 3 Department of Internal Medicine, General Hospital of Karditsa, Karditsa, GRC; 4 Department of Emergency Medicine, University of Thessaly, University General Hospital of Larissa, Larissa, GRC

**Keywords:** anti-cd20, covid 19, dyspnea, fever, immunocompromised patient, non hodgkin's lymphoma, obinutuzumab, post-covid symptoms

## Abstract

Persistent fever and pulmonary infiltrates in immunocompromised patients pose a major diagnostic challenge, especially in those receiving anti-CD20 therapy that impairs humoral immunity. We present a 66-year-old man with non-Hodgkin lymphoma on obinutuzumab and lenalidomide who developed prolonged fever, dry cough, and bilateral ground-glass opacities after COVID-19 infection. The differential diagnosis included persistent viral pneumonitis, post-COVID organizing pneumonia, drug-induced lung injury, opportunistic infection, and lymphoma progression. Initial corticosteroid therapy for suspected organizing pneumonia led to partial improvement, but symptoms relapsed during tapering. Bronchoscopy and laboratory analysis confirmed invasive pulmonary aspergillosis and profound hypogammaglobulinemia, while persistent SARS-CoV-2 PCR positivity suggested impaired viral clearance due to B-cell depletion. Treatment with isavuconazole, broad-spectrum antibiotics, corticosteroids, and intravenous immunoglobulin resulted in clinical, radiological, and immunological recovery. This case highlights the importance of maintaining a broad differential diagnosis in immunocompromised patients with unresolved post-COVID pulmonary symptoms and demonstrates that prompt recognition of hypogammaglobulinemia and timely immunoglobulin replacement can be pivotal for recovery.

## Introduction

Fever of unknown origin in immunocompromised individuals, particularly those with hematologic malignancies undergoing B-cell depleting therapy, presents a complex diagnostic challenge. Anti-CD20 agents, such as obinutuzumab, are widely used in the treatment of non-Hodgkin lymphoma and other B-cell neoplasms. While effective, they significantly impair humoral immunity, the antibody-mediated component of the immune system, leading to prolonged B-cell depletion, hypogammaglobulinemia, and increased vulnerability to infections [1,2].

The COVID-19 pandemic has further complicated the clinical landscape for this patient population. Persistent SARS-CoV-2 infection and delayed viral clearance have been frequently observed in individuals treated with anti-CD20 monoclonal antibodies, even after mild initial illness [3,4]. These patients often exhibit prolonged symptoms and test positivity, a phenomenon thought to arise from impaired viral clearance due to B-cell and T-cell exhaustion [5,6].

Furthermore, differentiating between various causes of prolonged respiratory symptoms and radiological findings in such patients is particularly challenging. Differential diagnoses include post-COVID organizing pneumonia (PCOP), persistent viral inflammation, drug-induced lung injury, and opportunistic infections, such as invasive pulmonary aspergillosis (IPA) [7,8]. PCOP is an inflammatory reaction of the small airways and alveoli that typically presents with bilateral ground-glass opacities, seen as hazy areas on CT scans that allow underlying structures, such as blood vessels, to remain visible, together with respiratory symptoms several weeks after initial COVID-19 recovery, and is often treated with corticosteroids [9,10]. However, radiological and clinical features frequently overlap with IPA and drug-related pneumonitis, making invasive diagnostics like bronchoscopy essential in persistent cases [11]. Finally, drug-induced lung injury should also be considered. Agents such as lenalidomide and obinutuzumab have been associated with interstitial lung disease and pneumonitis, forms of non-infectious lung inflammation caused by medication toxicity, that may mimic infectious or inflammatory etiologies [12,13].

These overlapping syndromes often coexist in the same patient, requiring a multidisciplinary diagnostic approach and timely therapeutic decisions. This case highlights how thorough immunologic evaluation, microbiologic diagnostics, and attention to therapy-induced complications are essential in guiding care.

## Case presentation

A 66-year-old non-smoker male with a history of non-Hodgkin lymphoma, diagnosed a decade earlier and treated with obinutuzumab and lenalidomide for the last three years, presented for evaluation of persistent fever, worsening respiratory symptoms, and bilateral pulmonary infiltrates unresponsive to outpatient treatment.

**Figure 1:**
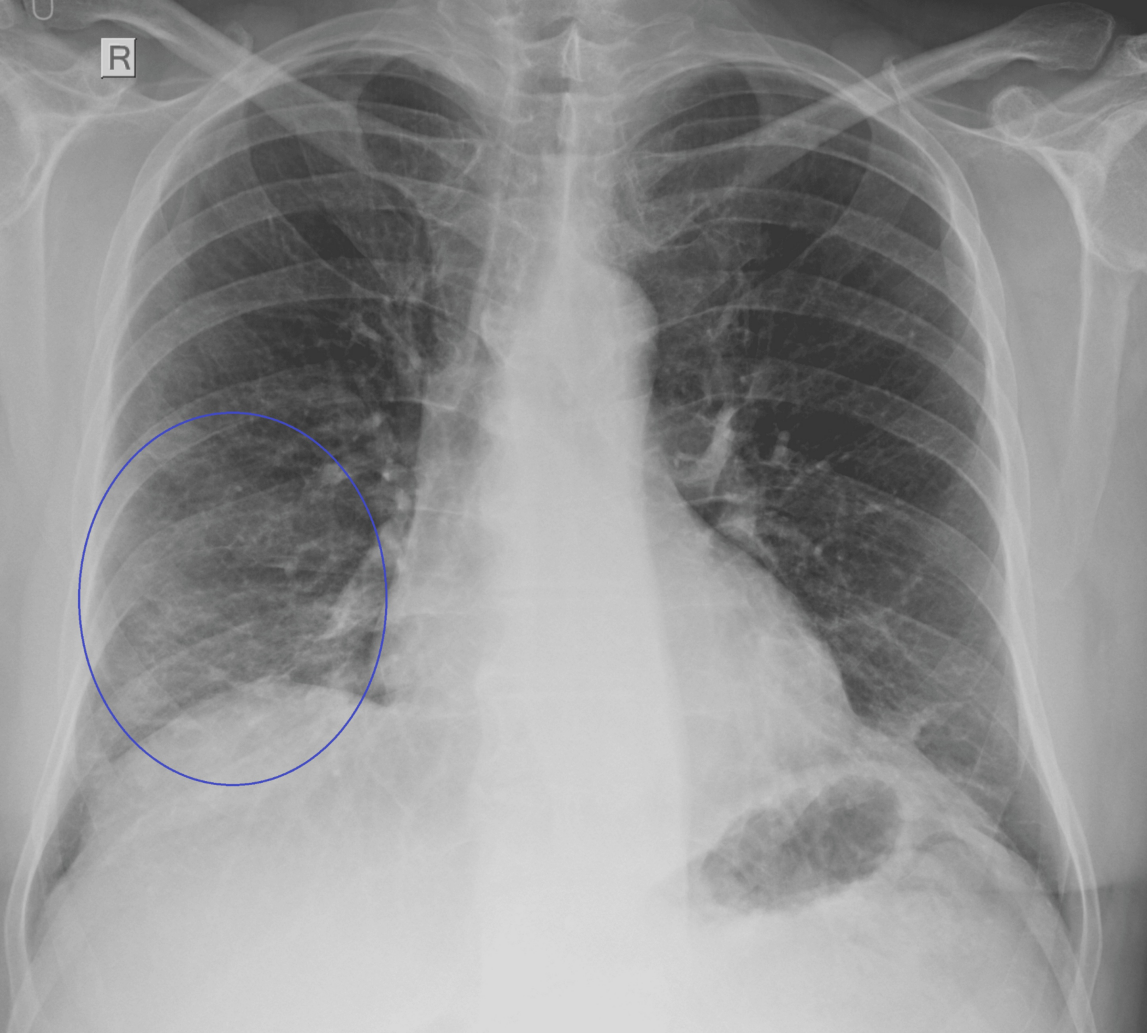
Chest X-ray at the onset of respiratory symptoms. Patchy ground-glass opacities are visible in the right mid-to-lower lung field and are indicated by a blue circle. Mild bilateral interlobular septal thickening is also present but is too subtle to be clearly delineated on plain radiography.

Approximately eight months prior to admission, he tested positive for SARS-CoV-2 at home. Chemotherapy was temporarily paused, and he completed a course of nirmatrelvir/ritonavir. Two months later, following a negative rapid antigen test and unremarkable chest X-ray, chemotherapy was resumed. Shortly thereafter, he developed a new cough and fever with a new X-ray showing patchy ground-glass opacities (Figure 1). 

**Figure 2:**
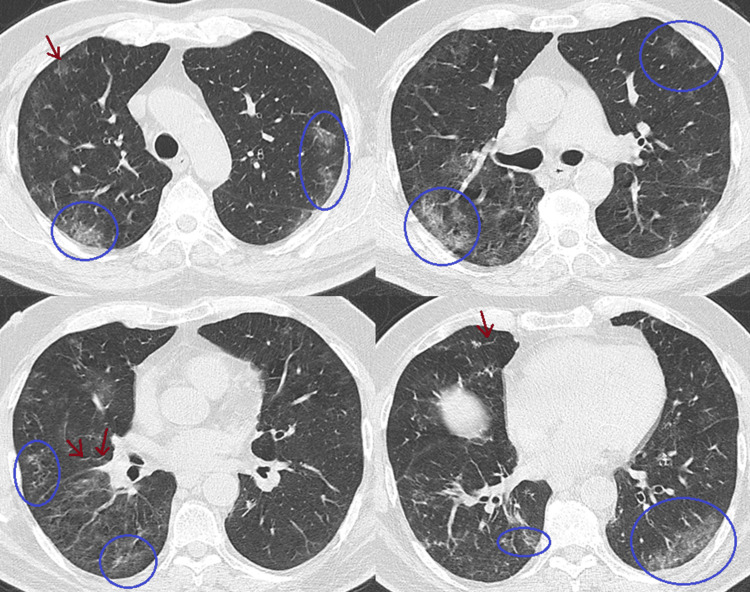
Chest computed tomography (CT) scan showing multiple bilateral peripheral ground-glass opacities (GGOs), predominantly in the lower lobes. GGOs are highlighted with blue circles. Mild interlobular septal thickening is also subtly present, indicated by red arrows.

He was empirically treated by his general practitioner as community-acquired pneumonia with moxifloxacin and inhaled corticosteroids. Repeat SARS-CoV-2 testing returned positive, and a chest computed tomography (CT) revealed bilateral peripheral ground-glass opacities (Figure 2), for which his general practitioner initially decided to observe conservatively. 

**Figure 3:**
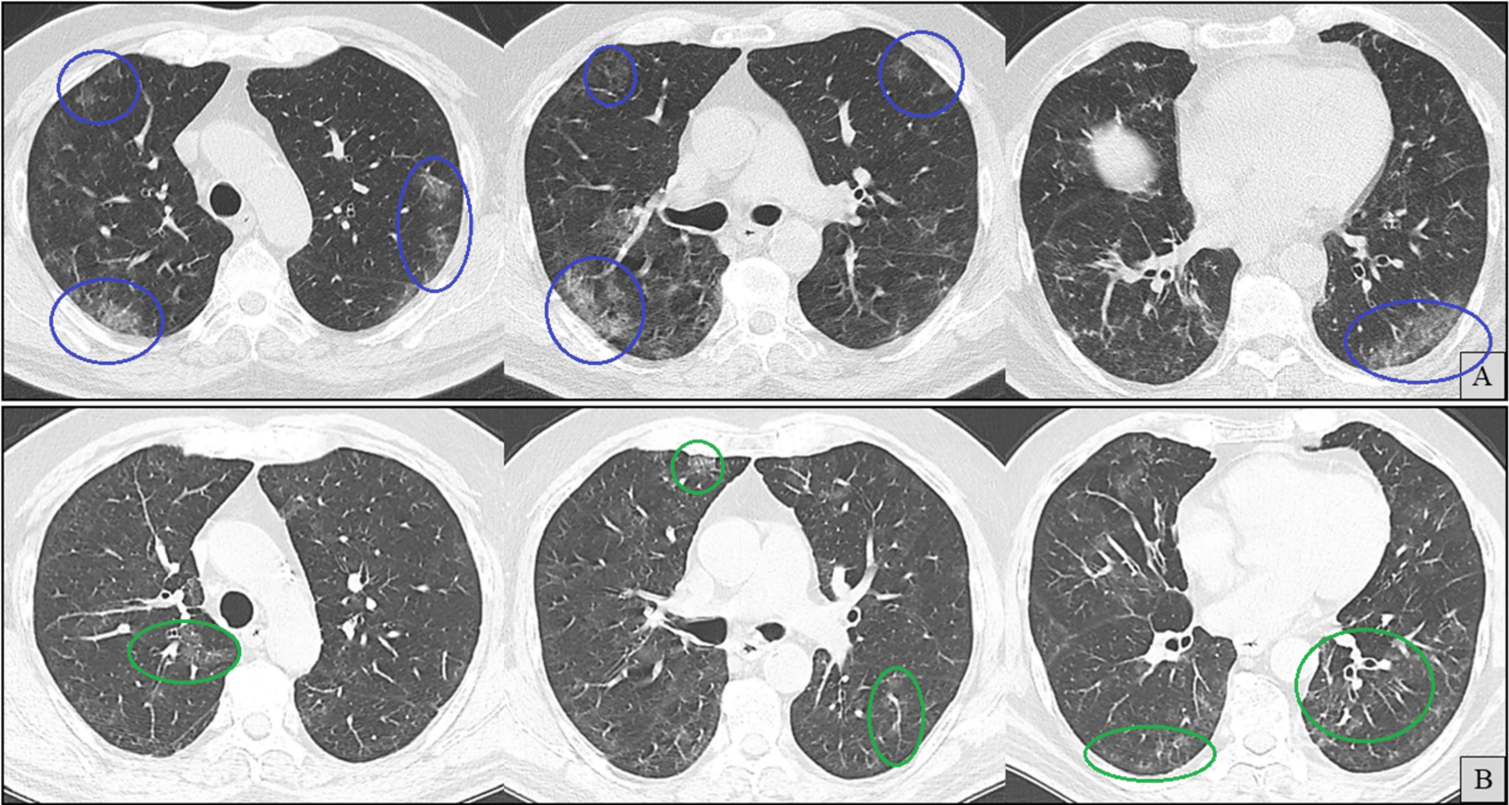
Axial chest computed tomography (CT) images (A and B). At the same anatomical levels, 20 days apart, with A (previously shown in Figure 2) included for comparison. B shows newly developed ground-glass opacities (green circles) and resolution of previous ones (blue circles), suggesting a possible migratory pattern.

With ongoing fever and cough for over 20 days, a follow-up CT (Figure 3) demonstrated no significant improvement in overall parenchymal involvement, although a migratory pattern of the ground-glass opacities was considered possible.

**Figure 4:**
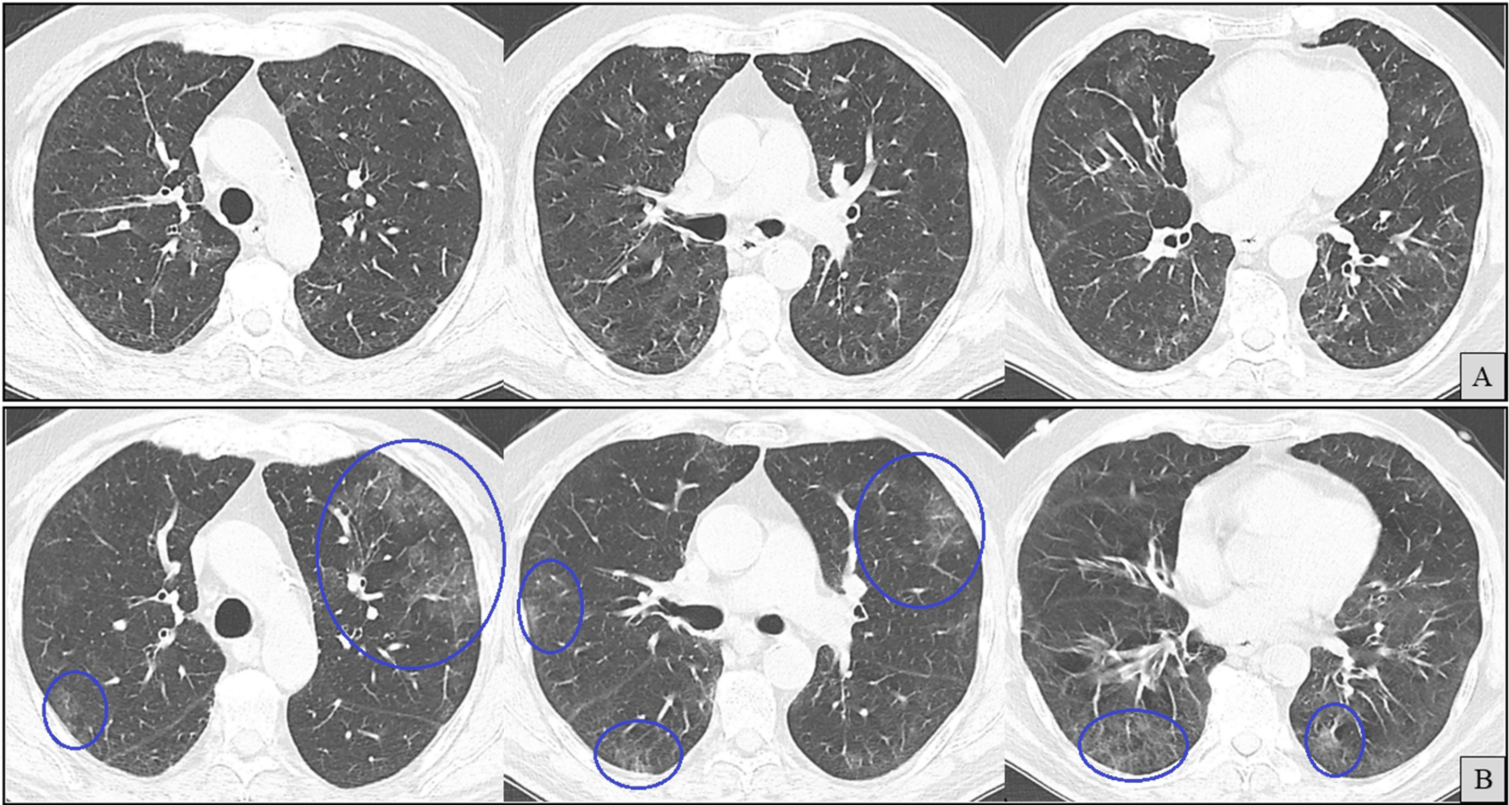
Axial chest computed tomography (CT) images (A and B) at the same anatomical levels, three months apart, with A (previously shown in Figure 3) included for comparison. B shows newly developed ground-glass opacities (blue circles).

Outpatient oral prednisolone (40 mg/day) was subsequently initiated for suspected PCOP-like inflammation. Symptoms partially improved, and over the next three months, the dose was gradually tapered to 15 mg/day. However, within days of reaching this lower dose, the patient experienced a relapse of dry cough and worsening radiographic findings (Figure 4).

**Figure 5:**
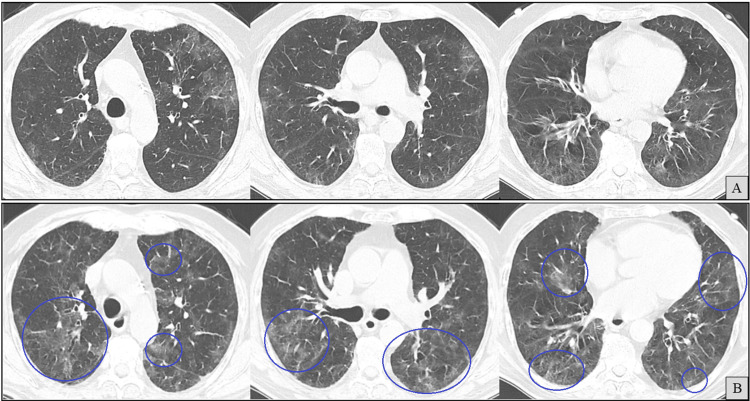
Axial chest computed tomography (CT) images (A and B) at the same anatomical levels, two months apart, with A (previously shown in Figure 4) included for comparison. B shows newly developed ground-glass opacities (blue circles).

Prednisolone was increased back to 40 mg/day, with plans for a more gradual taper. During this time, SARS-CoV-2 rapid antigen tests remained intermittently positive. After completing this two-month taper and while maintained on 25 mg/day of prednisolone, the patient again developed progressive dyspnea, low-grade fever, and exertional desaturation, approximately two months prior to admission. With a new CT scan showing further deterioration (Figure 5), a diagnostic bronchoscopy was performed.

Cytology from bronchoalveolar lavage (BAL) showed 49% lymphocytes with a markedly reduced CD4:CD8 ratio (0.1). Although no fungal elements were observed on cytology, BAL culture grew *Aspergillus fumigatus* complex, and PCR was also positive. Galactomannan from BAL was elevated (index: 2.0), whereas serum galactomannan and 1,3-β-D-glucan were negative. Pneumocystis testing was negative. The bronchoscopy findings are presented in Table 1.

**Table 1:** Bronchoscopy findings compatible with lymphocytic alveolitis and aspergillosis. AFB: acid-fast bacilli; BAL: bronchoalveolar lavage; CD: cluster of differentiation; CMV: cytomegalovirus; DFA: direct fluorescent antibody; HSV: herpes simplex virus; MTB: Mycobacterium tuberculosis; NK: natural killer cells; NKT: natural killer T cells; PCR: polymerase chain reaction; PMNs: polymorphonuclear neutrophils; SARS-CoV-2: severe acute respiratory syndrome coronavirus 2 ^a^The CD4:CD8 ratio reflects the balance between helper and cytotoxic T-cell subsets. ^b^Galactomannan is a fungal antigen marker used in the diagnosis of Aspergillus infection.

Parameter	Result/Value	Interpretation
BAL appearance	Turbid	Suggestive of inflammation/infection
Cytology	49% lymphocytes, CD4:CD8 ratio 0.1	Lymphocytic alveolitis
Cytology for malignancy	Negative	No malignant cells
Flow cytometry (BAL)		
- CD3+ T cells	99.5% of lymphocytes	Predominantly T cells
- CD4+ T cells	8.3%	Low helper T cell proportion
- CD8+ T cells	90.7%	Cytotoxic T cell dominance
- CD4:CD8 ratio^a^	0.1	Markedly reduced
- CD19+ B cells	0.2%	Low B-cell presence
- NK cells	1.2%	Low
- NKT cells	2.6%	Minor population
- Monocytes (CD14+)	3.1%	Present
- Neutrophils (PMNs)	15.2%	Mild neutrophilic component
- Eosinophils	0%	Absent
- Macrophages/epithelial cells	32.7%	Normal resident cells
FilmArray (BioFire Diagnostics, Salt Lake City, UT) (BAL)	Positive for SARS-CoV-2 only	No bacterial/viral co-infections detected
Bacterial culture	Negative	No growth
Fungal culture	Aspergillus fumigatus complex	Positive
Aspergillus PCR	Positive	Confirms Aspergillus DNA
Mycobacterial PCR (MTB)	Negative	No MTB DNA detected
AFB stain	Negative	No acid-fast bacilli
Galactomannan (BAL)^b^	Index 2.0	Elevated
Galactomannan (serum)^b^	Negative	No systemic antigenemia
1,3-β-D-glucan (serum)	Negative	No invasive fungal infection
*Pneumocystis jirovecii*PCR/DFA	Negative	Excluded
HSV/CMV PCR (BAL)	Negative	No herpesvirus co-infection

These findings raised concern for subacute IPA, although the overlapping steroid exposure, imaging, and clinical course also suggested organizing pneumonia or persistent viral inflammation. Quantitative immunoglobulin testing revealed profound hypogammaglobulinemia: total IgG was 105 mg/dL (normal: 847-1690), with all IgG subclasses markedly depressed. IgA and IgM levels were also undetectable. The complete results of the immunological assessment are presented in Table 2.

**Table 2:** Summary of immunological and serological evaluation, revealing profound hypogammaglobulinemia with absent IgA and IgM, markedly reduced IgG subclasses, and negative autoimmune and viral serologies. ANA: antinuclear antibodies; ANCA: antineutrophil cytoplasmic antibodies; Anti-HBc: hepatitis B core antibody; Anti-HBs: hepatitis B surface antibody; CCP: cyclic citrullinated peptide; C3/C4: complement components 3 and 4; HBsAg: hepatitis B surface antigen; HCV: hepatitis C virus; HIV: human immunodeficiency virus; Ig: immunoglobulin; MPO: myeloperoxidase; PR3: proteinase 3; RF: rheumatoid factor

Parameter	Result	Normal Range	Interpretation
IgG	105 mg/dL	847-1690 mg/dL	Profound hypogammaglobulinemia
IgA	<26.10 mg/dL	70-400 mg/dL	Undetectable
IgM	<18.60 mg/dL	40-230 mg/dL	Undetectable
IgG1	65 mg/dL	382-928 mg/dL	Markedly reduced
IgG2	35 mg/dL	241-700 mg/dL	Markedly reduced
IgG3	7 mg/dL	22-176 mg/dL	Low
IgG4	4 mg/dL	4-86 mg/dL	Low
RF	<9.38 mg/dL	<20 IU/mL	Negative
C3	128 mg/dL	90-180 mg/dL	Normal
C4	48 mg/dL	10-40 mg/dL	Elevated
ANTI-CCP IgG	<1 IU/mL	<7 IU/mL	Negative
ANTI-MPO ANCA	<2 IU/mL	<3.4 IU/mL	Negative
ANTI-PR3 ANCA	<2 IU/mL	<2.0 IU/mL	Negative
p-ANCA	Negative	Negative	Negative
c-ANCA	Negative	Negative	Negative
ANA	Negative	Negative	Negative
HBsAg	Negative	Negative	No evidence of HBV surface antigen
Anti-HBc	Negative	Negative	No prior HBV infection
Anti-HBs	2.29 IU/L	>10 IU/L	Non-immune
Anti-HCV	Negative	Negative	No evidence of HCV
Anti-HIV	Negative	Negative	No evidence of HIV

**Figure 6:**
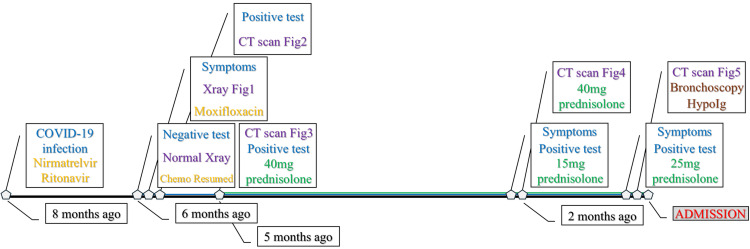
Timeline summarizing clinical events, treatments, imaging findings, and corticosteroid courses from COVID-19 infection to hospital admission. Color coding: COVID-19, clinical symptoms, SARS-CoV-2 tests (blue); main events before admission (brown); antiviral/antibiotics/chemotherapy (orange); chest imaging (purple); prednisolone doses (green). CT: computed tomography; Ig: immunoglobulin

The patient had a history of cumulative immunosuppression from B-cell depletion and corticosteroid exposure, placing him at risk for persistent viral infection and impaired pathogen clearance. The timeline depicting disease onset, radiological examinations, episodes of clinical deterioration, and corticosteroid tapering leading up to admission is presented next (Figure 6).

He was admitted to the respiratory ward with oxygen saturation of 93% on a 2 L nasal cannula and a Horowitz index of 214. Physical examination revealed bilateral inspiratory crackles; there were no signs of extrapulmonary involvement or peripheral lymphadenopathy. A summary of vital signs and laboratory findings at admission is shown in Table 3.

**Table 3:** Summary of vital signs and laboratory findings at admission. ALT: alanine aminotransferase; AST: aspartate aminotransferase; BNP: B-type natriuretic peptide; bpm: beats per minute; CRP: C-reactive protein; FiO₂: fraction of inspired oxygen; g/dL: grams per deciliter; HGB: hemoglobin; LDH: lactate dehydrogenase; LY%: lymphocyte percentage; mmHg: millimeters of mercury; NC: nasal cannula; NE%: neutrophil percentage; PaCO₂: partial pressure of carbon dioxide; PaO₂: partial pressure of oxygen; PLT: platelet count; SpO₂: peripheral oxygen saturation; U/L: units per liter; WBC: white blood cell count; ng/mL: nanograms per milliliter

Parameter	Result	Normal Range	Interpretation
Temperature	36.8°C	36.5-37.5°C	Normal
Blood pressure	120/75 mmHg	90-140/60-90 mmHg	Normal
Heart rate	102 bpm	60-100 bpm	Slightly elevated
Respiratory rate	19 breaths/minute	12-20 breaths/minute	Normal
SpO₂	93% on 2 L NC	>94%	Mild hypoxia
PaO₂	60 mmHg	>75 mmHg	Hypoxemia
PaCO₂	36 mmHg	35-45 mmHg	Normal
PaO₂/FiO₂ (Horowitz index)	214	>300	Impaired oxygenation
PaO₂/PaCO₂ ratio	1.66	Expected >2	Low
WBC	9.9 × 10⁹/L	4.5-10.5	Normal
HGB	11.9 g/dL	14.0-18.0	Mild anemia
PLT	332 × 10⁹/L	150-440	Normal
NE%	84.6%	40.0-70.0	Neutrophilia
LY%	4.7%	20.0-45.0	Lymphopenia
CRP	45 mg/L	<5 mg/L	Slightly elevated
Ferritin	1250 ng/mL	30-400	Elevated
LDH	257 U/L	135-225	Elevated
AST	15 U/L	0-40	Normal
ALT	11 U/L	0-40	Normal
Creatinine	0.74 mg/dL	0.72-1.20	Normal
Albumin	3.7 g/dL	3.5-5.0	Normal
BNP	75 pg/mL	<100	Normal
Procalcitonin	0.08 ng/mL	<0.1	Normal

**Figure 7:**
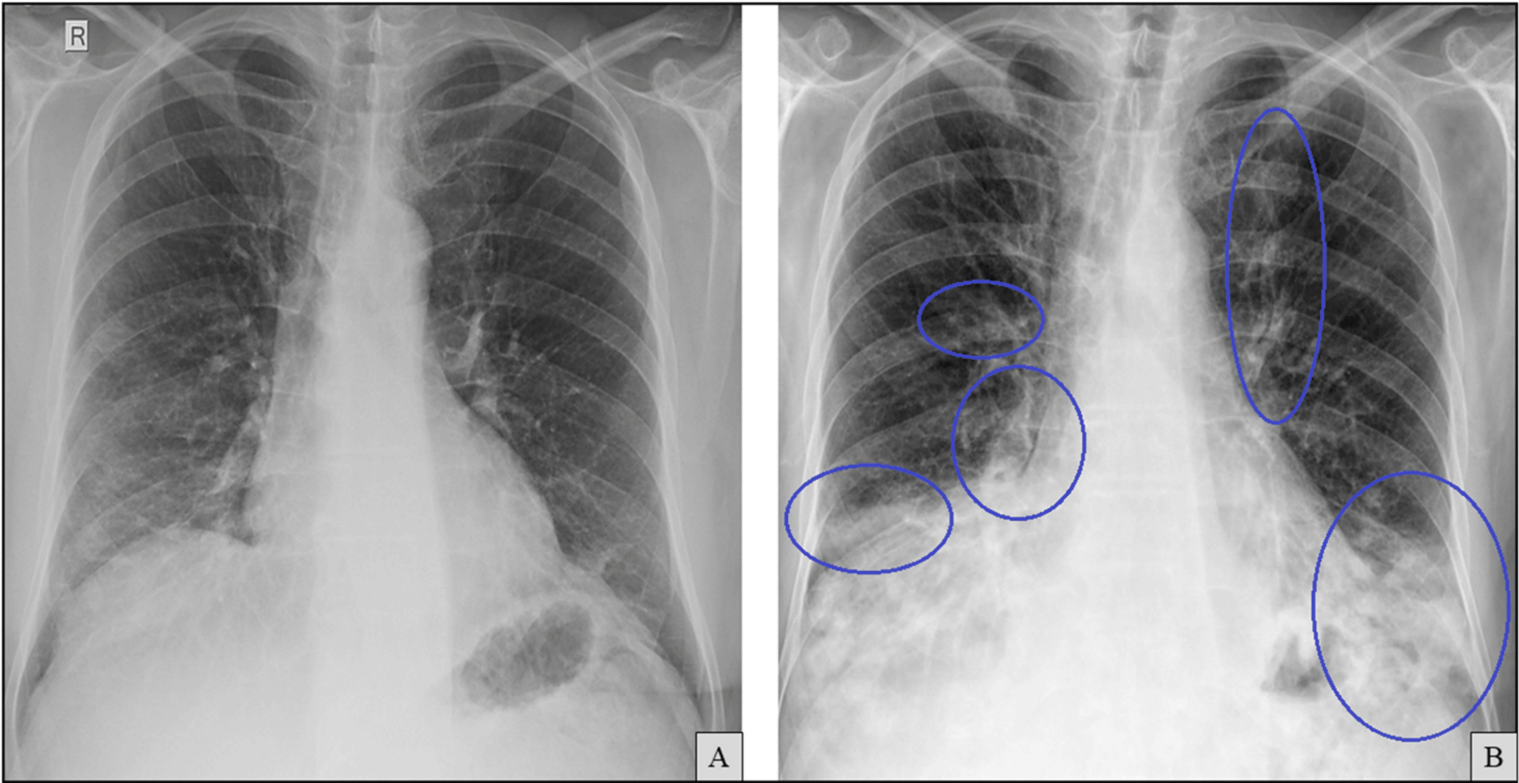
Chest X-ray at admission (B) showing worsening bilateral alveolar infiltrates compared to a previous film obtained seven months earlier (A, previously shown in Figure 1). Areas of radiological worsening are indicated by blue circles.

Chest radiography at admission demonstrated worsening bilateral alveolar infiltrates compared to imaging from seven months earlier (Figure 7).

**Figure 8:**
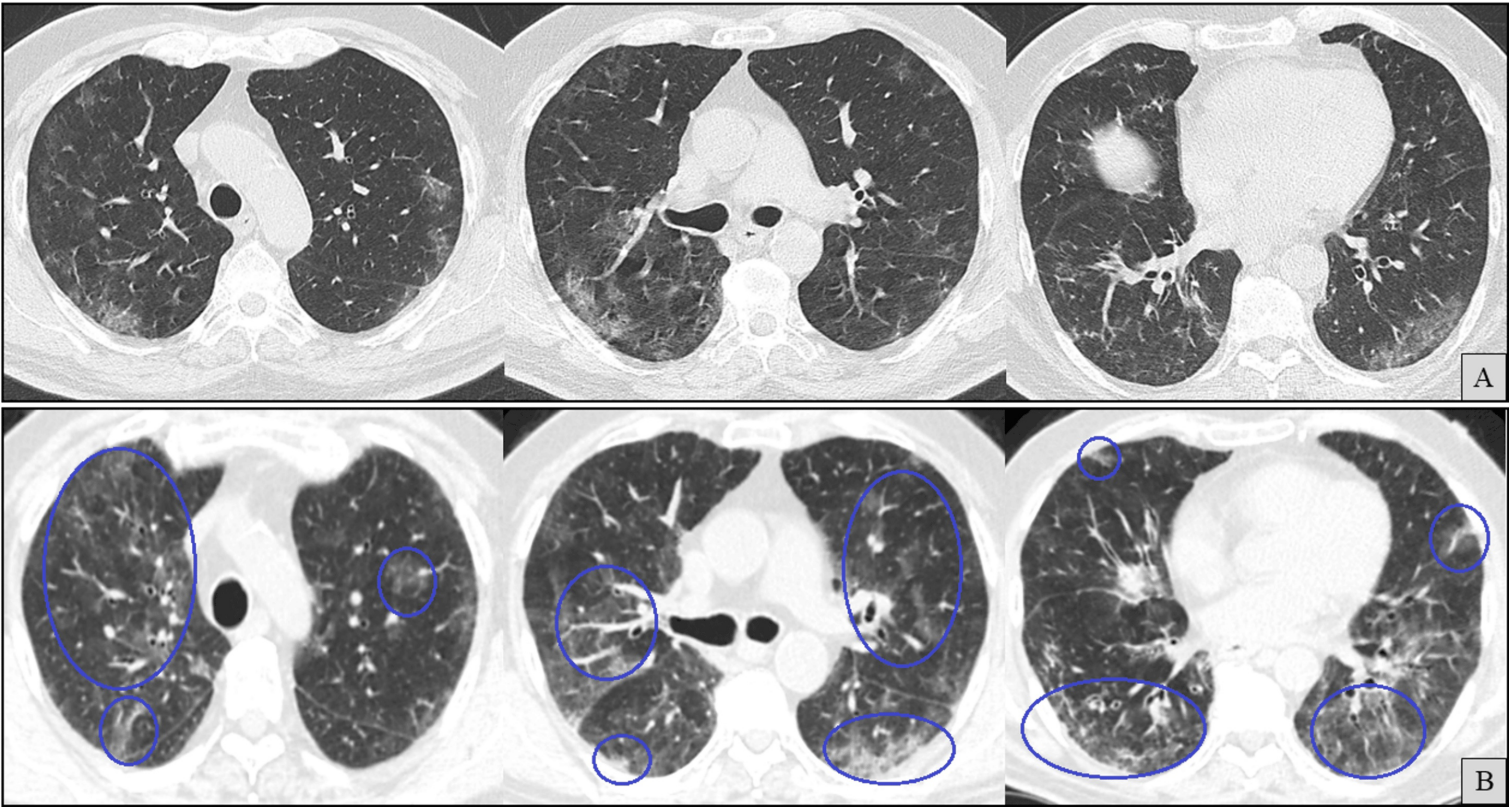
Axial chest computed tomography (CT) images (A and B) at the same anatomical levels, seven months apart, with A (previously shown in Figure 2) included for comparison. B shows newly developed ground-glass opacities (blue circles).

High-resolution CT at admission (Figure 8) revealed worsening patchy peripheral bilateral ground-glass opacities. No fibrotic features, such as traction bronchiectasis or honeycombing, were observed, and there were no pleural effusions.

**Figure 9:**
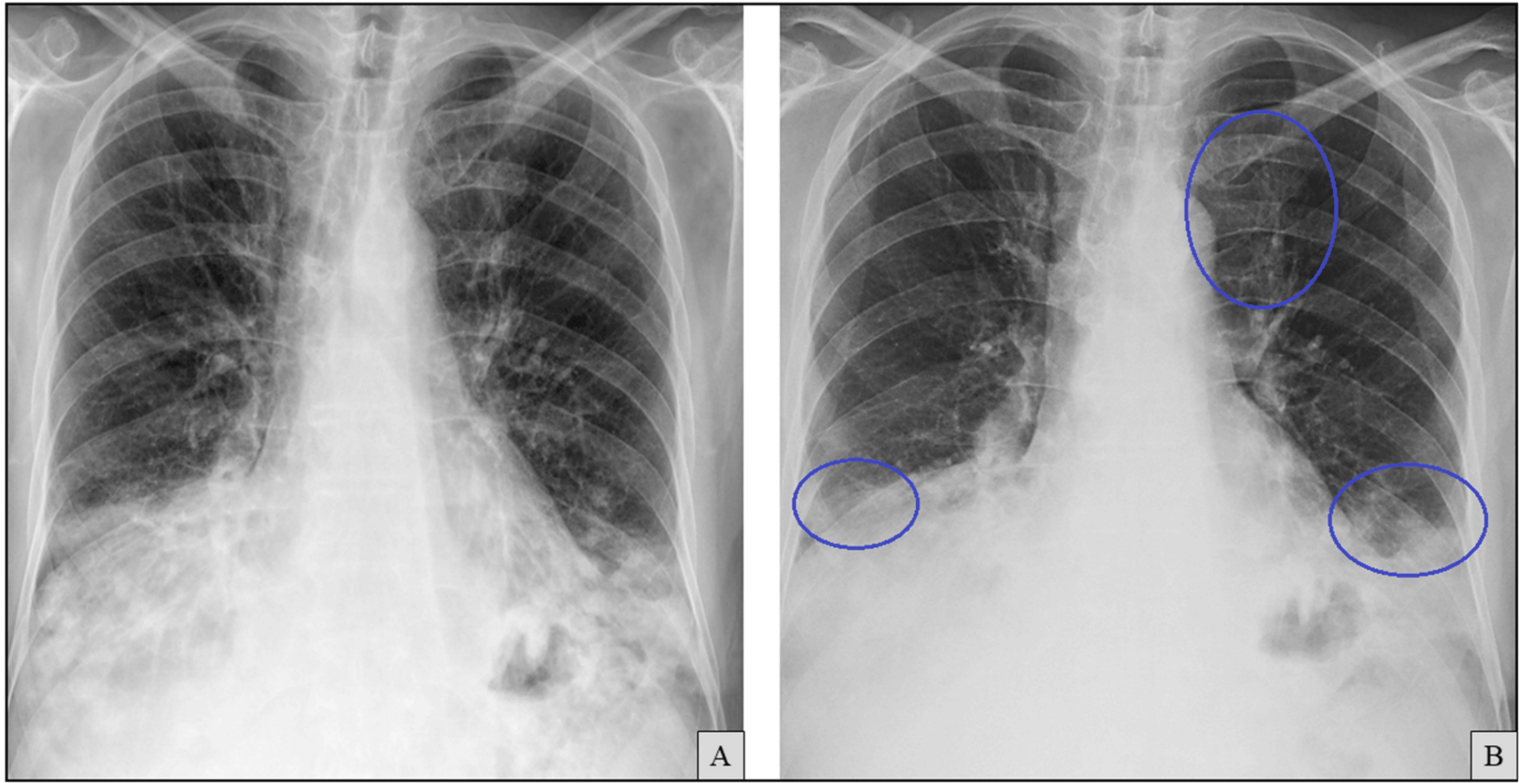
Chest X-ray at discharge (B) showing improvement of bilateral alveolar infiltrates compared to the film obtained at admission (A, previously shown in Figure 7). Areas of radiological improvement are indicated by blue circles.

A working diagnosis of persistent SARS-CoV-2 pneumonitis, probable subacute IPA, and organizing pneumonia-like inflammation was made, all in the setting of secondary hypogammaglobulinemia due to anti-CD20 therapy. Empirical treatment was initiated with intravenous isavuconazole, continuation of low-dose corticosteroids (prednisolone 20 mg/day), broad-spectrum antibiotics with cefepime/moxifloxacin, and a total of 150 g of intravenous immunoglobulin (IVIG, 2 g/kg). The patient also completed a 10-day course of remdesivir. Oxygen requirements were resolved rapidly, and the patient was weaned off supplemental oxygen by the second day of hospitalization. Inflammatory markers decreased, and chest film X-ray showed mild improvement (Figure 9); therefore, he was discharged after 10 days on tapering corticosteroids (prednisolone 15 mg/day at discharge) and oral isavuconazole.

**Figure 10:**
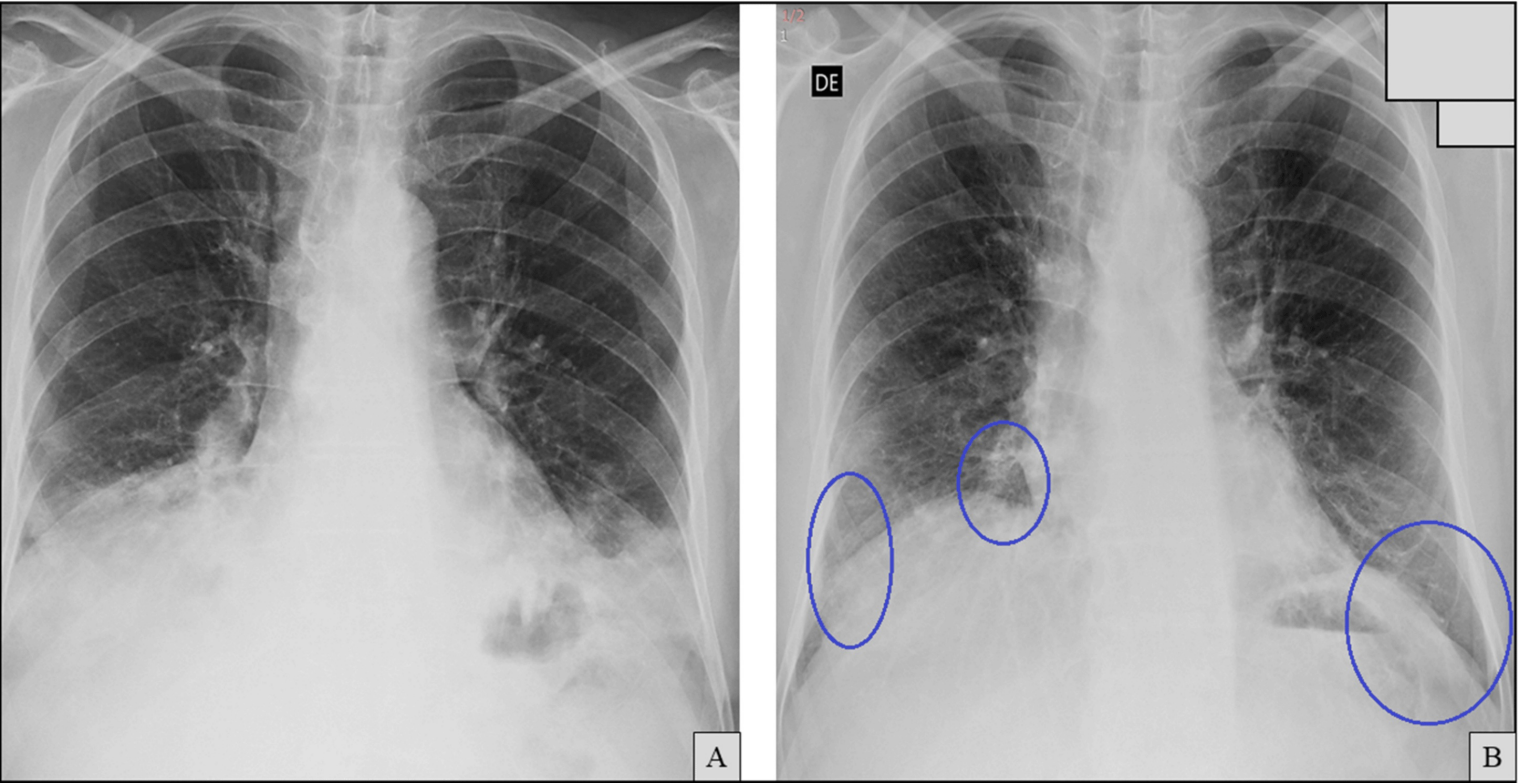
Chest X-ray at the one-month follow-up appointment (B) showing further improvement of bilateral alveolar infiltrates compared to the film obtained at discharge (A, previously shown in Figure 9). Areas of radiological improvement are indicated by blue circles.

After discharge, steroids continued to be tapered. At one-month follow-up, the patient remained asymptomatic on 5 mg prednisolone, and chest imaging showed near-complete resolution of infiltrates (Figure 10). However, total IgG was again reduced (733 mg/dL), prompting a second course of IVIG (100 g).

**Figure 11:**
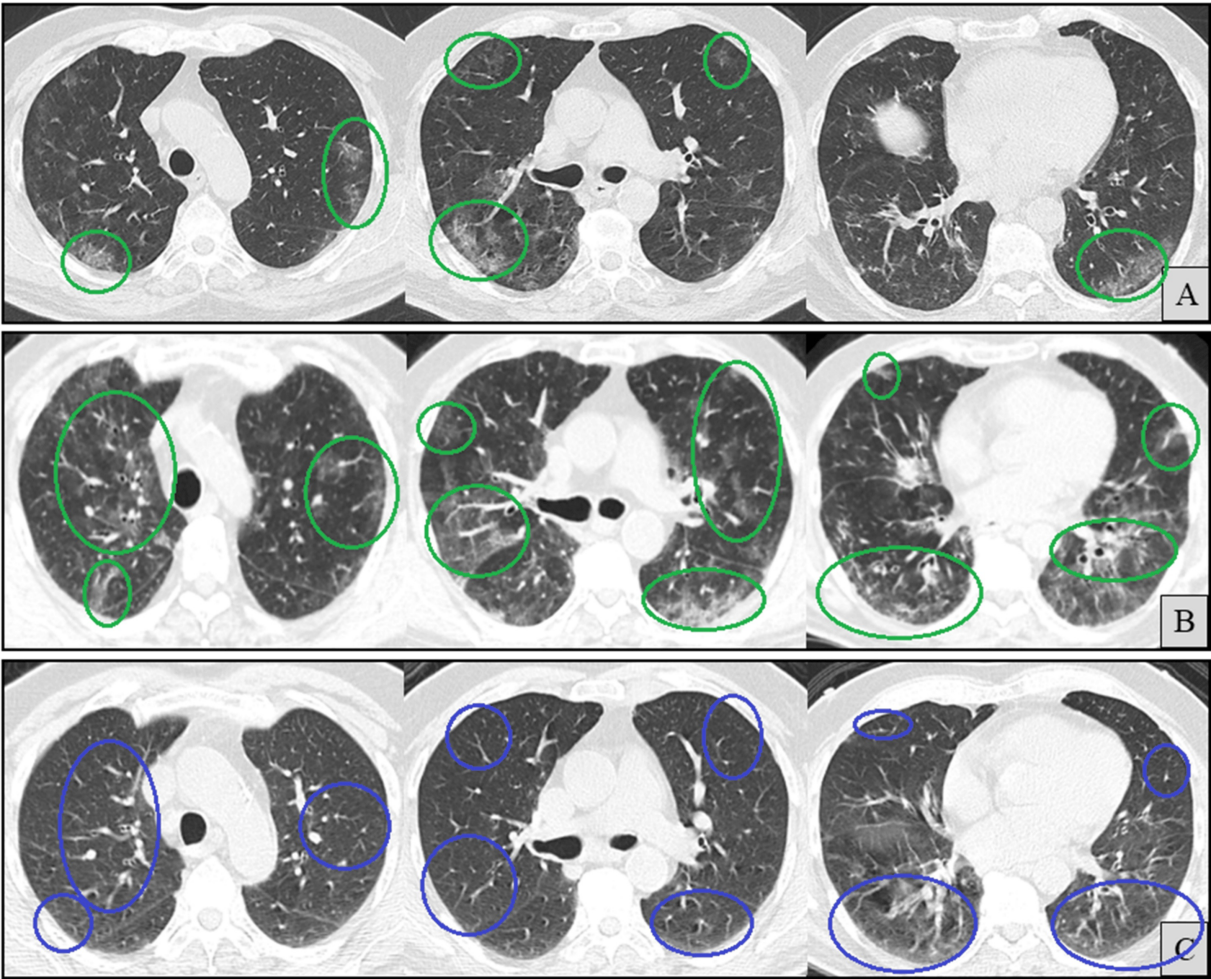
Axial chest computed tomography (CT) images (A-C) at the same anatomical levels. Taken nine months apart from A to C and two months apart from B to C. A (previously shown in Figure 2) and B (previously shown in Figure 8) are included for comparison. C demonstrates radiological improvement (blue circles) in areas previously showing ground-glass opacities (green circles).

At the two-month follow-up, corticosteroids were successfully discontinued, and serum IgG had normalized (876 mg/dL), including IgG1 and IgG2 subclasses; therefore, no further IVIG was administered. Repeat SARS-CoV-2 PCR testing was finally negative. Isavuconazole was also discontinued at that time, based on sustained clinical and radiological improvement (Figure 11). Respiratory symptoms did not recur, and corticosteroid-induced myopathy was noted but managed conservatively.

**Figure 12:**
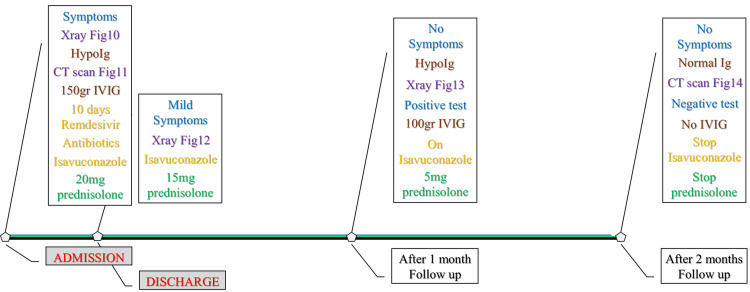
Timeline summarizing clinical symptoms, treatments, imaging, and corticosteroid tapering from hospital admission to two-month follow-up. Color coding: clinical symptoms, SARS-CoV-2 tests (blue); imaging (purple); immunoglobulin-related events (brown); medications (orange); prednisolone doses (green). CT: computed tomography; Ig: immunoglobulin; IVIG: intravenous immunoglobulin

The complete admission and post-admission timeline, including chest imaging findings, immunoglobulin levels, corticosteroid tapering, antifungal treatment, and IVIG dosing, is illustrated in Figure 12.

## Discussion

This case illustrates the complexity of managing persistent fever and pulmonary infiltrates in immunocompromised patients.

Initially managed as PCOP, the clinical course evolved to reveal opportunistic infection and severe immune dysregulation. PCOP is characterized by patchy consolidations and interstitial inflammation, typically responsive to corticosteroids [9,10]. The patient’s initial improvement with prednisone supported this working diagnosis, but relapse on dose reduction prompted reevaluation. The coexisting use of anti-CD20 agents and lenalidomide compounded immune suppression. Both agents impair B-cell function and contribute to humoral immunodeficiency, while corticosteroids further inhibit innate immune defenses [1,2,14]. The development of IPA, confirmed via BAL and positive PCR, is consistent with these risk factors. IPA must be considered in all cases of prolonged pulmonary symptoms in hematologic patients, particularly when imaging is nonresolving or atypical [11,15]. Persistent SARS-CoV-2 positivity throughout the treatment course highlighted another layer of complexity. Anti-CD20 therapy is associated with delayed viral clearance due to poor antibody generation and T-cell exhaustion [3,4,5]. Extended viremia and prolonged respiratory symptoms have been documented even in vaccinated patients treated with obinutuzumab [16]. In addition, drug-induced lung injury remained part of the differential. Lenalidomide has been associated with interstitial lung disease and organizing pneumonia patterns, and obinutuzumab has been implicated in noninfectious pneumonitis [12,13].

The overlapping features between drug toxicity, viral inflammation, and fungal infection required a comprehensive workup with imaging, bronchoscopy, and immunologic evaluation. A pivotal moment in this case was the recognition of severe hypogammaglobulinemia. Quantitative testing revealed profound IgG deficiency, likely secondary to years of B-cell depleting therapies [17]. In such patients, IVIG replacement is not only beneficial in preventing recurrent infections but can also contribute to immune modulation and viral clearance [18,19]. The administration of IVIG, alongside antifungal therapy and corticosteroids, resulted in rapid clinical and radiologic improvement. Follow-up imaging showed resolution of infiltrates, and normalization of IgG subclasses supported durable immune reconstitution.

In patients with multiple overlapping risk factors, such as immunosuppressive therapy, persistent viral presence, and prior COVID-19 infection, distinguishing between infectious and non-infectious inflammatory lung diseases, including organizing pneumonia, remains a significant clinical challenge [20]. Therefore, this case reinforces the importance of evaluating immunoglobulin levels in patients receiving anti-CD20 therapy who develop recurrent or prolonged symptomatology. Immunoglobulin replacement may offer significant benefit in treating both infectious and non-infectious inflammatory lung diseases and could be considered empirically in appropriately selected immunocompromised patients, given its favorable safety profile.

## Conclusions

This case underscores the diagnostic and therapeutic challenges in managing persistent respiratory symptoms in immunocompromised patients, particularly those undergoing anti-CD20-based regimens. Persistent fever and pulmonary opacities may reflect overlapping etiologies, including PCOP, opportunistic infections like invasive aspergillosis, and drug-induced lung injury.

Evaluation of immune status, especially immunoglobulin levels, is critical for guiding therapy. Our patient demonstrated substantial improvement after targeted antifungal treatment and intravenous immunoglobulin replacement, emphasizing the need for a multidisciplinary and immunologically informed approach to care in this high-risk population.
